# Physical performance in basic functional tests in premenopausal, perimenopausal and post-menopausal women

**DOI:** 10.17159/2078-516X/2025/v37i1a19964

**Published:** 2025-07-15

**Authors:** H Hove, A Zulu, N Khoza, L Smith

**Affiliations:** Department of Sport and Movement Studies, Faculty of Health Sciences, University of Johannesburg, Doornfontein Campus, Aukland Park, Johannesburg, South Africa

**Keywords:** women’s health, menopause, quality-of-life

## Abstract

**Background:**

Understanding the hormonal fluctuations and changes in the musculoskeletal system during menopause is important for health promotion and improving quality of life.

**Objectives:**

This study aimed to determine the physical ability of sedentary premenopausal, perimenopausal, and post-menopausal women.

**Methods:**

Female participants between the ages of 37 and 65 years (n=53) were divided into three groups according to menopausal transition guidelines and underwent anthropometric and physical tests. Descriptive and inferential statistics were computed, including *post-hoc* pairwise comparisons and ANOVA for significance between the three groups (p<0.05). The Bonferroni correction for multiple tests was applied. Results were expressed as mean and standard deviation.

**Results:**

Statistically significant differences were observed in weight (0.010) and waist circumference (0.0001) among the three groups. For the timed up-and-go test, premenopausal women performed significantly better than both perimenopausal (p=0.027) and postmenopausal women (p=0.0001). The one-minute push-up test showed a significant reduction in upper body strength from premenopause to postmenopause (p=0.002). The one-minute sit-up test showed significant declines between premenopause and postmenopause (p=0.001) and between perimenopause and postmenopause (p=0.030). The single-leg balance showed significant impairments in postmenopausal women compared to premenopausal women (p≤0.008 for both legs), and the sit-to-stand test revealed significant differences between premenopause and postmenopause (p=0.0001) and perimenopause and postmenopause (p=0.025).

**Conclusion:**

Premenopausal women exhibited the highest *p*-value significance and mean scores in various parameters, followed by the perimenopausal and post-menopausal groups. Physical performance in basic muscle function tests suggests a decline in muscle strength and endurance during the menopausal transition.

Female sex hormones significantly influence women’s health and well-being. Women aged between 37 and 65 experience profound hormonal changes due to ovarian ageing and the associated onset of menopause changes.[1,2] The menopausal transition phase is characterised by increased serum follicle-stimulating hormone concentration and decreased oestradiol concentration, which varies greatly between individuals.

Hormonal changes begin about five years before the onset of menopause and continue for years after the onset of menopause.[1] With age, muscle mass deteriorates, increasing the probability of muscular weakness later in life.[2] Menopause-related hormonal alterations, particularly in oestrogen levels, are typically associated with the loss of bone mineral density.[2] Reduction in muscle strength with oestrogen deficiency is not solely a consequence of reduced muscle mass but also because of a decrease in force of concentration per unit of muscle mass. This is partially explained by a loss of oestrogen-induced phosphorylation of the myosin regulatory light chain required for optimal force production of the myosin chain during cross-bridge cycling.[2] In addition, oestrogen is known to positively influence neural firing and motor unit activity[3], which could also explain the loss of muscle strength with ageing.[4,5] Evidence shows that oestrogen deprivation is associated with increased apoptotic activity in skeletal muscle, possibly related to reduced mitochondrial function and altered heat shock protein activity.[2]

Physical function may be affected by social and economic adversity throughout one’s life. It may also be shaped by a reproductive history characterised by giving birth at a very young age.[6] However, there is a significant gap in the literature, as few studies compare the menopausal phases and physical performance. This study aimed to compare physical performance in basic tests amongst premenopausal, perimenopausal, and postmenopausal women. This provides insight into the interplay between hormonal changes brought about by different menopausal stages and the impact it has on the basic physical performance of women during the menopausal transition.

## Methods

### Study design and participants

A cross-sectional, quantitative research design was used.

Non-proportional quota sampling was used to determine the desired sample size. The study recruited a total sample size of 53 participants - 18 in the premenopausal group, 22 in the perimenopausal group, and 13 in the postmenopausal group. Females between the ages of 37 years and 65 years from the University of Johannesburg Doornfontein Campus and the surrounding student residences were recruited through word of mouth to participate in the study.

Participants who did not meet the inclusion criteria were excluded from participation. The study did not exclude the use of hormonal contraceptives or hormonal replacement therapy. The exclusion criteria were as follows:

- Physically active lifestyle.
- Lactating and pregnant women.
- Self-reported smokers and potential participants with a BMI of >35kg/m^2^.
- Conditions that influence the functioning of the ovary, such as ovarian cancer, ovarian cysts, polycystic ovarian syndrome, and primary ovarian insufficiency.
- Chronic diseases and medicines that can result in muscle atrophy, such as Guillain-Barre syndrome, neural damage, amyotrophic lateral sclerosis, and spinal cord injury.
- Women who have had a hysterectomy.
- Women below the age of 37 years or above the age of 65 years.
- Participants with neurological impairments.
- Participants who have undergone a double oophorectomy.

### Ethical considerations

Ethical clearance was obtained from the Faculty of Health Sciences Research Ethics Committee at the University of Johannesburg (REC-1958–2023). Before testing, the objectives and testing procedures were explained to participants, and written informed consent was obtained from all participants.

## Testing procedures, measuring tools and instruments

The Stages of Reproductive Aging Workshop Classification scale (STRAW)[12,13] was used to determine the menopause stage. Menopause is classified into three stages: premenopause, perimenopause, and postmenopause. Premenopause, typically occurring from puberty until the early 40s, is characterised by regular menstrual cycles, stable hormone levels, and no significant menopausal symptoms. Perimenopause, generally beginning in the mid-40s, is marked by menstrual irregularities, fluctuating hormone levels, and symptoms such as hot flashes, night sweats, mood swings, sleep disturbances, vaginal dryness, and a decline in fertility.[13] During this phase, ovulation becomes less predictable, but pregnancy remains possible. Finally, postmenopause begins after 12 months of amenorrhea, usually around age 51 or older. In this stage, menstrual periods cease entirely, and while symptoms like hot flashes and vaginal dryness may persist, they tend to diminish over time.[13] This stage also carries increased risks for osteoporosis and cardiovascular diseases due to lower oestrogen levels, and fertility ends completely.[13] A limitation of the current study is that the ovarian stage was not confirmed by measuring circulating follicle-stimulating hormone concentrations.[13] Participants were characterised into the respective menopausal groups based on their menstrual cycle regulatory patterns, irregularities, or absence, which coincided with other symptoms defined by ovarian stage guidelines.

Anthropometric measurements, including height, weight, waist circumference, and body mass index, were taken to determine body composition. Physical tests were performed to evaluate muscular strength, muscle endurance, and physical performance (Table 1). The tests included the 30-second single-leg balance, 1-minute sit-up, 1-minute push-up, 5- repetition sit-to-stand, and timed up-and-go test.

### Statistical analysis

The data were analysed using the Statistical Package for the Social Sciences (SPSS, version 29.0) for Windows Version 15.0 (SPSS Inc., Chicago, IL, USA). Descriptive and inferential statistics were computed with a confidence level of 95% and a statistical significance threshold of p<0.05. The normality of the data was assessed using the Shapiro-Wilk and Kolmogorov-Smirnov tests. Post-hoc pairwise comparisons were conducted for the statistically significant findings to identify any differences among the calculated means of all possible pairs, with significance values adjusted by the Bonferroni correction for multiple tests to mitigate the increased risk of type I errors from performing multiple statistical tests. ANOVA was utilised to analyse the nominal data from the body composition and screening tests. To assess the significance of the differences in muscle strength and endurance across menopausal stages, the Tukey Honestly Significant Difference (HSD) test for multiple comparisons was performed.

## Results

### Demographic data

Table 2 displays the demographics of the sample of the 53 participants recruited for this study. The youngest participant was 37 years and the oldest was 65 years old. The mean age was calculated at 47.3 years across all menopausal stages. The difference in ages between all three groups, namely premenopause, perimenopause and postmenopause was statistically significant (p=0.001). The mean body mass was 79.2kg, with the values ranging from a minimum of 56.0 kg and a maximum of 99.0kg. There was no significant difference in body mass and waist circumference between premenopausal and perimenopausal women, with only a significant increase observed in body mass and waist circumference in postmenopausal women when compared to both premenopausal and perimenopausal women. This could imply that this change only takes place after the menopause transition. A statistically significant difference in weight was found among the three groups (p=0.01). The height ranged from 150.0 cm to 189.1cm and the sample had a mean height of 168.7 cm. The mean body mass index was 28.0kg/m^2^, ranging from 20.3kg/m^2^ to 35.1kg/m^2^. The mean waist circumference was 94.9cm, ranging from 73.0cm to 139.0cm. There was a significant difference (p=0.001) in waist circumference across the groups.

### Physical tests

The descriptive data are shown in Table 3. The premenopausal group had the best score for the timed up- and-go test with a mean score of 6.3 seconds, and the post-menopausal group (9.5 seconds) had the slowest time. For the one-minute push-up test, the premenopausal group (17.4 repetitions) scored the highest, and the postmenopausal group scored the lowest, with a mean of 7.7 repetitions. The scores of the one-minute sit-up test followed the same order, with the premenopausal group (23.8 repetitions) scoring the highest and the postmenopausal group (8.5 repetitions).

The data from the physical performance tests reveals a consistent decline in physical function across the menopausal transition, with significant differences observed among the three groups in most tests. In the timed up-and-go test, premenopausal women performed significantly better than both perimenopausal (p=0.027) and postmenopausal women (p=0.0001), indicating a progressive decline in mobility and dynamic balance. Similarly, the one-minute push-up test showed significantly reduced upper body strength from premenopause to postmenopause (p=0.002). However, the difference between perimenopause and postmenopause was not significant (p=0.147). This suggests that muscle strength declines rapidly during the early transition but may stabilise later. The one-minute sit-up test, which assesses core strength, demonstrated significant declines between premenopause and postmenopause (p=0.001) and between perimenopause and postmenopause (p=0.030), highlighting progressive weakness in abdominal muscles. Balance tests, such as the thirty-second single-leg balance, showed significant impairments in postmenopausal women compared to premenopausal women (p≤0.008 for both legs), reflecting reduced proprioception and neuromuscular control. Lastly, the five-rep sit-to-stand test, which assesses lower body strength, revealed significant differences between premenopause and postmenopause (p=0.0001) and between perimenopause and postmenopause (p=0.025), indicating a decline in functional capacity. These findings collectively underline the physiological impact of the menopausal transition, marked by reduced muscle strength, balance, and mobility due to hormonal changes, sarcopenia, and potential declines in physical activity. The results emphasise the importance of early interventions, such as strength and balance training, to mitigate these functional losses and maintain quality of life during and after menopause.

## Discussion

This study compared physical performance in basic muscle strength tests amongst premenopausal, perimenopausal, and post-menopausal women.

The mean age of the perimenopausal group was 48.4 years. This is in line with a similar study in America which stated the mean age for perimenopausal stage as 47 years.[15] Socioeconomic factors, geographical factors, dietary habits, reproductive factors, body composition, level of physical activity, and history of tobacco usage influence the differences in the age of menopause onset.[16] The current study found that the most notable change in indices of body fat gain (body mass and waist circumference) occurred after the menopausal transition. All three menopausal groups would be considered overweight based on the mean body mass index of >25 kg/m^2^. The three groups also yielded scores above the norm for waist circumference (>88cm). These results highlight the increased prevalence of abdominal obesity in the menopausal population. Similar results were reproduced in a study on obesity and menopause in which the onset of menopause coincided with increased obesity.[17] Although total body fat percentage was reported as a better measure of obesity than body mass index amongst the menopausal population, the same study identified the trend of increased body mass index and body fat percentage during the menopausal transition.[18]

Premenopausal women consistently demonstrated higher performance levels in the various muscle strength and endurance tests than perimenopausal and postmenopausal women. They showed a significantly better performance or a tendency for better performance in selected tests compared with perimenopausal women in this study’s sample. An association between muscle strength and endurance was noted, as the participants who performed better in the strength test also performed well in the endurance test. These findings align with a study that emphasised the role of muscle strength in improving muscular endurance.[19] Their research observed significant improvements in muscular endurance with enhanced muscle strength.[19] Similar findings were reported in another study, which showed that the decline in muscle strength and lean muscle mass during the menopausal transition resulted in decreased muscular endurance.[20] This trend in muscle strength and endurance can be attributed to fluctuations in sex hormones. Oestrogen plays a vital role in maintaining muscle mass and function. A recent study suggested that oestrogen levels in perimenopausal women remain relatively stable compared to the substantial decline observed in post-menopausal women.[21] This hormonal stability may explain the higher muscle strength and endurance observed in perimenopausal women, as oestrogen’s protective effects on muscle tissue are better preserved.

The findings in this study indicate that premenopausal women yielded better physical performance scores than their perimenopausal and post-menopausal counterparts. Notably, for the Timed up-and-go test, premenopausal women performed significantly better than perimenopausal (p=0.027) and postmenopausal women (p=0.0001), indicating a progressive decline in mobility and dynamic balance. Similarly, a study examined physical performance across the menopausal stages and reported significant declines in postmenopausal women, consistent with the findings in this study.[22] The five reps sit-to-stand test showed significant differences between premenopause and postmenopause (p=0.0001) and between perimenopause and postmenopause (p=0.025), indicating a decline in functional capacity. These findings collectively underline the physiological impact of the menopausal transition, marked by reduced muscle strength, balance, and mobility due to hormonal changes, sarcopenia, and potential declines in physical activity.

The higher results scored for physical performance amongst the premenopausal group can be attributed to hormonal stability, which helps preserve muscle function and mobility.[21] The higher oestrogen levels in the premenopausal stage likely contribute to better coordination, agility, and overall physical capability. In addition, oestrogen is known to positively influence neural firing and motor unit activity[3] and ageing, which could also explain the loss of muscle strength with ageing.[4,5]

### Limitations

The researchers note the following limitations in this study:

- Cross-Sectional Design: This study adopted a cross-sectional research design, which prevented the researchers from recording the longitudinal changes that may occur over extended periods.
- Limited test variety: The selected tests provided a limited perspective on muscle strength and physical performance.
- Sample size: The study involved 53 participants, which may not represent the entire menopause population in this age range.
- Complex interactions: This study did not consider the factors influencing musculoskeletal health and physical performance, namely genetics, lifestyle, and comorbidities.

### Recommendations for future research

The laboratory tests can determine participants’ menopausal status and assess the relationship between hormonal changes and basic muscle function. It is challenging to study the effects of the menopausal transition in isolation from regular ageing, so a study that can isolate and evaluate the effects of the menopausal transition alone is recommended.

## Conclusion

This study provides evidence that premenopausal women exhibit higher muscle strength, endurance, and physical performance in basic functional tasks than perimenopausal and postmenopausal women. The results suggest that hormonal changes due to menopause lead to a decline in muscle strength, endurance, and overall physical performance. These findings align with previous research, suggesting that hormonal stability, particularly for oestrogen levels, is crucial in maintaining musculoskeletal health during the menopausal transition. Understanding these dynamics is essential for tailoring exercise and healthcare interventions to optimise the health and well-being of women as they navigate the challenges of menopause.

## Figures and Tables

**Table 1:** The physical tests were categorised as strength, endurance, and physical performance tests

Test	Purpose	Protocol	Scoring	Equipment
Timed up- and-go test[7]	The purpose of this test is to assess agility.	Participants began in a seated position with their hands at their sides. They were instructed to rise from the chair, walk three metres, turn around the cone, walk back, and sit down again as quickly as safely possible. The timer started when they were told to get up and stopped when they sat back down.	Participants were scored based on the speed of their completion of the task test.	
30-second single leg balance test[8]	The purpose of this test was to assess postural steadiness and balance.	On a flat surface, the participant began with both feet on the ground. They then lifted one foot, attempting to maintain this position for 30 seconds without hooking it behind the standing leg or touching it to the ground.	The participants were scored based on the duration, in seconds, that they maintained the single-leg stance.	
1-minute sit-up test[9]	The purpose of this test was to assess abdominal and hip flexor muscle strength and endurance.	Participants began in a supine position on the exercise mat, with their knees bent at 90° and feet flat on the ground. They were then instructed to lift their head and trunk off the mat towards their knees without using their hands. This movement was repeated as many times as possible within one minute.	The participants were evaluated based on the number of repetitions they could perform with proper form within 60 seconds.	Exercise mat Stop watch Clipboard Pen
1-minute push-up test[10]	The purpose of this test was to assess upper limb muscle strength and endurance	Participants began in a plank position, with slight hip flexion and their knees on the ground. They were then instructed to repeatedly lower their chin and chest to hover just above the exercise mat and then return to the starting position as many times as possible in 1 minute.	The participants were scored on the number of repetitions they were able to complete with good form within 60 seconds.	Exercise mat Stop watch Clipboard Pen
5-repetition sit-to-stand test[11]	The purpose of this test is to assess lower body functional strength	Participants began in a seated position with their arms crossed over their chest and feet flat on the ground. They were then asked to stand up from the chair and sit back down five times, as quickly as possible, without using their hands for assistance.	Participants were scored based on the time, in seconds, it takes them to complete 5 repetitions.	Chair with backrest Stop watch Clipboard Pen

**Table 2:** Demographic data comprising age, weight, height, body mass index and waist circumference (n=53)

				95% Confidence Interval For Mean		Range		Sig. between groups

		N	Mean±SD	Lower Bound	Upper Bound	Min	Max	

Age (years)	Premenopause	18	40.6±3.6	38.9	42.4	37	51	
	Perimenopause	22	48.4±5.8	45.8	50.9	37	57	
	Postmenopause	13	54.5±6.6	50.6	58.5	44	65	

	Total	53	47.3±7.5	45.2	49.3	37	65	0.00*

Body mass (kg)	Premenopause	18	76.5±7.3	72.9	80.1	64.0	95.0	
	Perimenopause	22	77.8±9.4	73.6	81.9	56.0	98.0	
	Postmenopause	13	85.5±7.0	81.2	89.7	75.0	99.0	

	Total	53	79.2±8.8	76.8	81.7	56.0	99.0	0.01*

Height (cm)	Premenopause	18	168.4±6.9	164.9	171.8	153.0	183.0	
	Perimenopause	22	168.1±8.7	164.3	171.9	150.0	185.2	
	Postmenopause	13	169.9±9.8	164.0	175.9	160.0	189.1	

	Total	53	168.7±8.3	166.4	170.9	150.0	189.1	0.81

Body mass index (kg/m^2^)	Premenopause	18	27.2±3.6	25.4	28.9	21.6	34.2	
	Perimenopause	22	27.7±3.5	26.1	29.3	20.3	35.1	
	Postmenopause	13	29.6±3.9	27.3	31.9	22.9	34.7	

	Total	53	27.9±3.7	26.9	29.0	20.3	35.1	0.17

Waist circumference (cm)	Premenopause	18	88.3±9.6	83.5	93.0	73.0	110.0	
	Perimenopause	22	93.4±11.2	88.4	98.4	74.3	118.0	
	Post-menopause	13	106.4±15.1	97.3	115.6	85.0	139.0	

	Total	53	94.9±13.5	91.1	98.6	73.0	139.0	0.00*

* Indicates significance p<0.05

**Table 3:** Descriptive data from the physical tests (n=53)

				95% Confidence Interval For Mean		Range		Sig. between groups

		N	Mean±SD	Lower Bound	Upper Bound	Min	Max	
Timed up-and-go test (seconds)	Premenopause	18	6.3±1.2	5.67	6.9	4.6	10.1	peri: 0.027 post: 0.000
	Perimenopause	22	8.0±1.7	7.24	8.8	5.1	13.0	pre: 0.027 post: 0.092
	Postmenopause	22	9.5±3.2	7.59	11.5	6.1	17.3	pre: 0.000 peri: 0.092

	Total	53	7.8±2.4	7.13	8.4	4.6	17.3	0.085 *

1-Minute push-up test (repetitions)	Premenopause	18	17.4±7.2	13.85	21.0	0	26	peri: 0.104 post: 0.002
	Perimenopause	22	12.6±7.1	9.43	15.8	0	24	pre: 0.104 post: 0.147
	Postmenopause	13	7.7±7.8	2.96	12.4	0	23	pre: 0.002 peri: 0.147

	Total	53	13.0±8.1	10.80	15.3	0	26	0.144*

1-Minute sit up test (repetitions)	Premenopause	18	23.8±12.8	17.48	30.2	0	46	peri: 0.225 post: 0.001
	Perimenopause	22	18.2±9.3	14.12	22.3	1	38	post: 0.001 post: 0.030
	Post-menopause	13	8.5±8.9	3.12	13.9	0	31	pre: 0.001 peri: 0.030

	Total	53	17.8±11.9	14.48	21.0	0	46	0.279*

30-Second left single-leg balance (seconds)	Premenopause	18	28.9±2.7	27.63	30.3	20.0	30.0	peri: 0.338 post: 0.002
	Perimenopause	22	26.3±5.2	24.05	28.6	12.0	30.0	pre: 0.3378 post: 0.040
	Post-menopause	13	21.3±9.5	15.72	26.8	0.0	30.0	pre: 0.002 peri: 0.040

	Total	53	25.9±6.4	24.21	27.8	0.0	30.0	0.396*

30-Second right single-leg balance (seconds)	Premenopause	18	29.3±1.7	28.47	30.2	23.0	30.0	peri: 0.695 post: 0.002
	Perimenopause	22	28.0±3.0	26.70	29.4	20.1	30.0	pre: 0.695 post: 0.008
	Post-menopause	13	22.7±9.0	17.18	28.1	0.0	30.0	pre: 0.002 peri: 0.008

	Total	53	27.1±5.5	25.64	28.7	0.0	30.0	0.734*

	Premenopause	18	7.1±1.4	6.45	7.9	4.4	10.0	peri: 0.111 post: 0.000
	Perimenopause	22	9.1±2.0	8.20	10.0	5.6	13.1	pre: 0.111 post: 0.025
	Post-menopause	13	11.9±5.2	8.8	15.1	7.6	23.5	pre: 0.000 peri: 0.025

	Total	53	9.1±3.4	8.2	10.1	4.4	23.5	1.000*

* Indicates significance p<0.05
